# Sustainable Landscaping and Sustainability Education on a College Campus: A Case Study in Place‐Based, Collaborative Ecosystem Science

**DOI:** 10.1002/ece3.74486

**Published:** 2026-10-11

**Authors:** Christine S. O'Connell, Shannon Hahn, Evelyn Jauregui, Michael V. Frazier, Anika R. Bratt, Mary A. Heskel, Megan Butler, Christie M. Manning

**Affiliations:** ^1^ Department of Environmental Studies Macalester College St. Paul Minnesota USA; ^2^ Schmid College of Science and Technology Chapman University Orange California USA; ^3^ Facilities Services Macalester College St. Paul Minnesota USA; ^4^ Department of Biology Macalester College St. Paul Minnesota USA; ^5^ Sustainability Office Macalester College St. Paul Minnesota USA

**Keywords:** campus land management, experiential learning, living laboratory, place‐based collaborative sustainability science, sustainable landscaping

## Abstract

Sustainability education on college campuses can leverage interdisciplinary collaborations between facilities services, sustainability offices, environmental studies researchers, and students to provide hands‐on opportunities for place‐based learning. In an effort led by student researchers that could be replicated in academic practice at other academic institutions, we collaborated across campus groups to improve campus landscaping and turfgrass management. Turfgrass is an ideal cover for high‐traffic areas and recreation, but is energy intensive to maintain, inefficient at carbon sequestration, and requires fertilizing which contributes to nutrient pollution. Here, we outline a student‐led research effort that combined experiential learning, using a college campus as a “living laboratory,” and an ecosystem services case study to advance not only campus sustainability goals but also provide a rigorous research opportunity for undergraduates. Supported by a collaborative, multi‐office team at Macalester College, students provided recommendations on campus land management practices that would increase a multi‐faceted suite of sustainability goals. Student researchers used geospatial data to measure campus land cover types then adapted an easily accessible ecosystem model, i‐Tree, to quantify changes to ecosystem services due to converting strategic parcels of lawn to native plant beds or trees. Based on model results, students proposed increasing Macalester's tree cover to 30% campus area and converting 6381 m^2^ of lawn to low‐input native gardens. These landscape changes were modeled to sequester 26.3 tons of carbon year^−1^ for a value of USD $521 year^−1^ and remove air pollutants, a health benefit valued at USD $1034 year^−1^. This proposal balanced campus climate and environmental sustainability goals, Facilities' labor and economic constraints, and landscaping use goals. This use case of a college campus as a “living laboratory” offers a novel case study that empowered undergraduates and can be adapted to similar campuses that integrate the work and expertise of multiple students, faculty, and staff.

## Graphical abstract

This article presents a case study in place‐based, collaborative ecosystem science at Macalester College. We describe a successful partnership between academic departments, the sustainability office, and facilities services to assess the benefits of converting campus turfgrass to native plantings. This work serves as a replicable model for bridging research and practice to achieve tangible sustainability goals on university campuses.

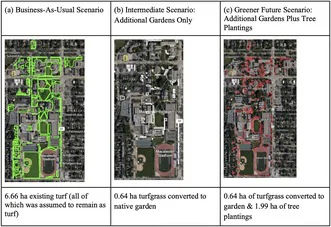

## 1 Introduction

Institutionally managed landscapes, encompassing the campuses of colleges, universities, hospitals, and similar entities, are prominent landscapes within urban environments and can account for large tracts of urban and suburban footprints (Li et al. 2014). While small in footprint compared to agricultural land use, urban and suburban landscapes have a disproportionate impact on ecosystem services and disservices as they are hotspots for nutrient and energy cycling (Liang and Gong 2020). Within this context, institutionally managed lands not only contribute to the esthetics of urban/suburban settings but are often managed by single, independently governed land managers who could have an outsized influence on the environmental and ecological footprints of urban and suburban landscapes (Čibik and Štěpánková 2020).

Of institutionally managed landscapes, land holdings managed by colleges and universities are beholden to myriad goals and missions. These goals could include providing recreation and beauty, promoting community spaces, fostering community engagement, providing experiential learning opportunities as a “living laboratory” (Gomez and Derr 2021), and helping the college or university meet any potential sustainability goals (Too and Bajracharya 2015; Posner and Stuart 2013). One challenge of marrying campus sustainability goals with the other strategic roles of campus landscapes is the widespread presence of managed turfgrass lawns. Turfgrass lawns are hearty and robust to foot traffic, have a general uniform appearance, bounce back after a wide range of weather conditions, and are spaces well suited to community events (Mathew 2021; Monteiro 2017). On many college campuses, their desirability is tied to ease of maintenance, the inherent need for turfgrass to support athletics infrastructure, and the usefulness of turfgrass for events such as reunion, commencement, and other outdoor events that take place in grass‐lined gathering areas, as well as a cultural and social component to wanting homogeneous, weedless, manicured lawns as a desired campus esthetic.

Turfgrass ecosystems provide ecosystem services, especially compared to impervious surfaces, however their environmental benefit by some metrics is limited (Mathew 2021; Monteiro 2017). For example, in one widely utilized household‐level urban carbon flux model, turfgrass ecosystems are estimated to sequester carbon for 75 years after planting, while other types of plants, e.g., native grasses, woody herbaceous species, or trees, sequester a larger amount of carbon over a similarly long or longer time period (Baker et al. 2007). The replacement of lawn cover by other species has the potential to impact the carbon cycle, the water cycle (including both water quantity and water quality), and air quality, in addition to other ecosystem services (Leung et al. 2011). Quantifying the changes to ecosystem services delivery when replacing turfgrass with other landscape coverage on institutionally‐managed lands such as college campuses is pivotal for informed environmental stewardship.

Here, we describe a case study in place‐based, collaborative ecosystem science that combined educational goals for undergraduate research trainees and sustainability goals related to the removal of turfgrass on a college campus, which could be replicated at other institutions of higher education. We convened a collaborative team comprising undergraduate student summer research fellows and members of the Macalester College Office of Sustainability, Facilities Services, and the Environmental Studies department to model and quantify the ecological benefits of replacing strategically chosen tracts of lawn with non‐lawn land coverage. This collaborative effort was led by undergraduate students who worked with all stakeholders to design their experiment and served for the involved staff and faculty as an opportunity to implement an experiential learning summer research experience for early‐career undergraduate environmental science students. Further, this work reflected realistic, actionable changes to the landscape management on Macalester's campus and took into account the labor and budgetary constraints and campus landscape goals beyond sustainability that also influence the presence of lawn vs. non‐lawn parcels. Using two easily‐accessible ecosystem models, iLawn and i‐Tree, we modeled two scenarios—a Business‐As‐Usual (BAU) scenario and a Greener Future (GR) scenario—and quantified the impact on carbon storage, air quality, and water cycling of replacing parcels of turfgrass with native plants and trees. In this manuscript, we outline the pedagogical and mentoring approach used in addition to the guided collaborative documents that we developed to share information between stakeholders in the Office of Sustainability and Facilities Services, which can serve as a toolkit for others pursuing similar on‐campus research efforts that center student learning.

Notably, students were motivated to address the historic role of lawns in the suburban American (white) idyll and in post‐WWII consumerist America (Steinberg 2006, 2013). The lawn chemical industry in the United States has been argued to have promoted turfgrass esthetics as a route to “healthy” communities despite the ecological impacts of that same proliferating turfgrass monoculture (Robbins and Sharp 2003). These social dynamics also intersect with race and class, with the potential for landscaping practices to “normalize white bourgeois ideas about taste, order, beauty, and land” (Lebowitz and Trudeau 2016). Participating student researchers hoped to decenter the importance of lawn on campus due to both the complicated relationship between lawn esthetics and social justice (Lang 2018; Lebowitz and Trudeau 2016; Robbins 2012), as well as Macalester's institutional sustainability goals. For those reasons, we further posit that similar collaborations elsewhere have the potential to advance not only sustainability goals on college campuses but also to tie into efforts to reimagine the residential college experience as a space for decolonized thinking (Hooks 2014).

### 1.1 The Campus as a “Living Laboratory” and Site of Experiential Learning

This case study serves as an easily‐replicable model of how interdisciplinary teams on a college campus can leverage Sustainability and Facilities infrastructure to create a “living laboratory” experiential learning research experience for undergraduate research students. Experiential learning, characterized by learning through direct engagement and practice (Browne et al. 2020; Favaloro et al. 2019), has been demonstrated to enhance students' perceived competence and personal development (Savage et al. 2015), support critical thinking skills (Heinrich et al. 2015), and increase feelings of preparedness for future sustainability initiatives in course‐based projects (Belkhir 2015). Among engineering students, such applied learning approaches in sustainability education have also improved interdisciplinary problem‐solving and the ability to address societal challenges (Sundman et al. 2025). These opportunities are often, but not always, delivered via coursework and with structured, curriculum‐associated learning goals (Davidson et al. 2021; Domask 2007). For instance, sustainability and environmental studies programs may implement experiential learning by engaging students in projects such as preparing sustainability reports for real‐world clients or stakeholders during a semester‐long course, fostering teamwork and applied skill development (Sulkowski et al. 2020).

Experiential learning can also utilize the college or university campus itself as a “living laboratory,” in which the institution's dynamic socio‐ecological landscape is utilized for practical, place‐based learning (Evans et al. 2015; Hansen 2016; Lindstrom and Middlecamp 2017; Save et al. 2021; Zen 2017). Examples include student farms at land‐grant universities (Parr and Trexler 2011), conservation areas utilized for field studies (Gunnels et al. 2021), and integration of campus‐generated data into curricula, such as chemistry case studies developed in collaboration with campus facilities management (Lindstrom and Middlecamp 2017). Numerous further opportunities exist to expand the role of campus living laboratories in academic practice and for the concurrent advancement of student professional development and campus sustainability goals (Evans et al. 2015; Hansen 2016; Zen 2017). Our case study here attempts to do so outside of the context of a traditional course and instead during a supported summer undergraduate research fellowship, a model that could be widely replicated on college campuses that prioritize undergraduate research experiences. Indeed, pedagogical objectives and deliverable goals were co‐designed by the entire team of stakeholders, including undergraduate research fellows, making this approach nimble and flexible to the needs of the practitioners, educators, and students.

### 1.2 Campus Context

Research was conducted on the campus of Macalester College, located in an urban residential area of St. Paul, Minnesota, USA (latitude, longitude: 44.93, −93.17). The ecosystem biome type can be characterized as Oak savanna and north central interior forest, with a total annual precipitation normal of 77.75 cm and mean annual temperature of 7.89°C (Bratt et al. 2017). The student body is approximately 2100. Sustainability is an institutional priority at Macalester: it was an early signatory to the Talloires Declaration, the first official statement made by university administrators to declare a commitment to environmental sustainability in higher education (Saleh et al. 2015; Wright 2002), and established a Sustainability Office in 2008.

Macalester maintains roughly 13.4 ha of grounds for a balance of esthetic purposes, ecosystem services, social utility, and educational goals (Table 1). In addition to these goals, Macalester's Sustainability Plan at the time of this project promoted the delivery of several ecosystem services: (a) achieve carbon neutrality, which can be achieved in part via landscape carbon sequestration; (b) infiltrate the first inch of rainwater during rain events, via rain gardens and other landscape management decisions; (c) increase biodiversity and pollinator habitat, which the college had begun by establishing several pollinator paths on campus and leaving a campus parcel in grassland; and (d) reduce energy costs and increase energy efficiency through passive solar heating. Additionally, Macalester holds as a general landscape goal to replace low‐quality grass patches with perennial flower beds and install additional rain and pollinator gardens.

**TABLE 1:** Summary of the four categories of goals that Macalester College has for its landscape.

Ecosystem services	Social utility	Esthetic purposes	Educational opportunities
Institutional sustainability goal at the time of the project: campus carbon neutrality (including via landscape carbon sequestration) Infiltrate the first inch of rainwater from each rainstorm (via rain gardens and permeable pavers) Increase biodiversity and pollinator habitat Reduce energy costs and increase energy efficiency through passive solar heating	Important greenspace for students Fields for recreation and sports Focal communal greenspace for the community Safety	Emphasize view of campus from the outside Maintain institutional visual tradition	Campus as a living learning laboratory (via prairie outdoor “classroom,” rain gardens, student engagement) Outdoor teaching spaces

### 1.3 Nutrient Cycling and Ecological Dynamics of Turfgrass vs. Sustainable Land Cover

As humans establish and manage turfgrass lawns, they change nutrient and water cycle dynamics, including the carbon (C), nitrogen (N) and phosphorus (P) cycles, the rate of water infiltration, the rate of evapotranspiration, and the rate of water runoff (Law and Patton 2017; Petrovic and Easton 2005; Stier et al. 2013; Hobbie et al. 2017; Walsh et al. 2005), which in turn alters ecosystem service delivery (Mathew 2021; Tong et al. 2015). The differences in ecosystem services provided by lawn versus non‐lawn land cover are also heavily influenced by landscape management interventions (e.g., differences in fertilizer use, irrigation, or whether gasoline‐powered mowers are used) (Barnes et al. 2020; Monteiro 2017).

Fertilizer application to lawns dramatically influences the N and P cycling on urban landscapes. For example, fertilizer application is the largest source of N pollution to urban watersheds in St. Paul, MN (Hobbie et al. 2017). The rate and timing of N loss from a system after fertilizer addition depend on numerous factors, including the form of fertilizer, the amount of fertilizer added, the timing of fertilizer addition, soil moisture, and the root depth of the species covering the landscape (Carey et al. 2012; Gerber et al. 2016; Cameron et al. 2013; Barton and Colmer 2006; Bowman et al. 2002). Runoff due to P fertilizer use has been associated in the northern hemisphere with algal blooms and eutrophication in lakes and waterways (Carpenter 2008). In the Twin Cities, fertilizer use has been found to be excessive (Baker et al. 2007). The Minnesota Phosphorus Lawn Fertilizer Law banned P fertilizer use on lawns in most contexts in the state of Minnesota. State bans on P fertilizer use are common in the US: over ten states have implemented similar regulations (Lee and McCann 2018). Both reducing turfgrass cover and landscape management can have measurable effects on the rate of N and P loss (Carey et al. 2012; Chen et al. 2023).

Ecosystem service delivery likely differs substantially between urban lawns and urban non‐lawn plant coverage. Turfgrass lawn generally stores less carbon in plant tissues than perennial plant beds and woodland/forest ecosystems (Lal and Augustin 2011), while soil organic carbon stocks may increase or hold steady after conversion of non‐turfgrass land uses to turfgrass (Qian and Follett 2002; Phillips et al. 2023). Trees and native perennial plants have also been associated with lower concentrations of air pollutants than turfgrass lawns, due to differences in leaf structure complexity, stomatal uptake rates, and increased deposition rates associated with larger leaf area indices (Nowak et al. 2006; Grote et al. 2016). These pollutants can include carbon monoxide (CO), nitrogen dioxide (NO_2_), ozone (O_3_), particulate matter of different diameters (PM10 and PM2.5), and sulfur dioxide (SO_2_). Different plant species remove air pollutants at different rates, with tree species contributing to higher rates of removal than lawn (Mathew 2021; Tong et al. 2015; Leung et al. 2011). Further, fertilizer and other inputs (e.g., pesticides, fungicides, etc.) and gas‐powered motors associated with lawn maintenance that are used at higher rates than in plant bed or tree maintenance can lead to the emission of air pollutants and carbon emissions (Harvey et al. 2014; Kong et al. 2014).

Urban environments create large disruptions to the water cycle. Impervious surfaces—i.e., surfaces which prevent water infiltration—lower soil water infiltration rates, increase runoff and overland water flow, and are associated with urban flooding and reduced rates of groundwater recharge (Walsh et al. 2005). Higher rates of overland flow in urban areas can also increase concentrations of pollutant transport into urban water bodies (Wan et al. 2023). In contrast, pervious surface coverage, including lawn, plant beds and woodland/forest cover, allow for soil water infiltration, reducing overland flow, and increase landscape evapotranspiration (ET) rates, which contribute to local atmospheric cooling (Qiu et al. 2013). Evapotranspiration rates differ across species, with turfgrass commonly measuring the lowest ET rates (Litvak et al. 2014).

### 1.4 Status Quo Landscaping Practices at Study Outset

On Macalester's main campus, irrigation, mowing/weeding, and fertilizer application are all a routine part of land management. Macalester fertilizes its lawn up to 5 times a year, depending in part on whether a lawn is used in a high‐performance sporting context. In 2020, Macalester used two fertilizer types: 9‐12‐6 NPK starter fertilizer and EndoRoots 3‐3‐4 NPK. Mowing is done 1–2 times per week during the growing season, and clippings are left where they fall on the turf. For weed and pest management, Macalester Facilities utilize a combination of integrated pest management (including manual removal of harmful weeds and bugs and biological control) and herbicides and insecticides when needed (applied as spot spray when damage is too great to remove by hand).

Macalester also maintains pollinator habitat in the form of several native species gardens, planted in conjunction with other local institutions and residents to form a neighborhood pollinator corridor. Water management includes multiple porous paver paths, and two rain gardens bordering one of the larger tracts of impervious surface parking, designed to reduce flooding and overland runoff as well as retain N and P.

## 2 Methods

### 2.1 Goal and Knowledge Integration Between On‐Campus Stakeholders

This project came about during the height of the 2020 COVID pandemic. Because campus was forced to close, lab‐based student research projects could not be completed as planned. We instead sought an outdoor‐based ecology/biology project for which data could be collected outside, analysis could be completed using already‐purchased equipment, and collaborative work could be done outside or online, while still allowing students to meet independent research pedagogical goals. In collaboration with the student summer research fellows, we quickly settled on landscaping as an ecologically relevant and practical research focus (Table 2). To begin the project, the faculty research advisor contacted the Director of Sustainability about sustainability goals that included landscaping. The Director of Sustainability shared the college landscaping plans, and in addition, brought in the Lead Groundsperson from Facilities. Through subsequent online and email conversation between the students, faculty research advisor, and Sustainability and Facilities staff members, it quickly became clear that there was a shared enthusiasm for exploring the possibilities of converting some turfgrass parcels on campus to native bed plant cover and/or trees.

**TABLE 2:** Summary of undergraduate student involvement, pedagogical objectives, and deliverables across project phases.

Project phase or activity	Student involvement and supporting mentors	Professional development/pedagogical goals	Product/deliverable
Project design and planning	Collaborative project co‐design by Sustainability Office staff, faculty mentor, and undergraduate research fellows	Developing research design skills; interdisciplinary collaboration; connecting theory and practice	Defined project scope and goals
Stakeholder interviews and engagement	Students planned and conducted interviews with Facilities and Sustainability teams; co‐mentored by staff and faculty	Communication and interview methods; stakeholder engagement; qualitative data collection	Tour and Interview Question Guide (Data S1)
Data collection and landscape mapping	Students conducted campus walking tours to identify potential planting and tree sites alongside Facilities mentor staff member; students led landscape classification using i‐Tree Canopy and development of BAU and GR scenarios	Technical skills in i‐Tree, a commonly used management tool; understanding campus ecosystem; designing landscape management scenarios	Geospatial maps (Figure 1)
Ecosystem services modeling	Students independently learned and applied i‐Tree modeling tools using online tutorials; mentored by faculty and staff	Technical skills in i‐Tree ecosystem services modeling; quantitative analysis; data visualization	Modeled estimates of ecosystem services under BAU and GR campus land management scenarios (Figures 2 and 3, and Table 3)
Data synthesis and report writing	Students synthesized quantitative and qualitative data; co‐mentored on writing and analysis	Research synthesis; professional writing in sustainability practice	Short‐format report for internal Sustainability Office use summarizing findings and campus landscaping recommendations
Presentation and dissemination	Students created academic posters for campus showcase and professional conference presentations; co‐mentored in poster design and during conference attendance	Professional poster design skills for an academic audience; oral presentation skills for an academic audience	Posters presented internally at Macalester College and at an Ecological Society of America annual meeting

The project's goals evolved to balance the different priorities of stakeholders across campus, a crucial step in the place‐based nature of this work, and an important step when considering the challenges that similar campus‐based interdisciplinary projects could potentially face. In this case, the project participants had to balance the needs of the Facilities Services team (e.g., campus esthetics, toughness/resilience of landscaping, ease of care), the faculty participants (e.g., consideration for the scholarly and pedagogical value of the project, ecological and ecosystem services outcomes), the student participants (e.g., a project that aligns with their sustainability values and furthers their learning goals), and the Office of Sustainability team (e.g., meeting campus‐wide sustainability goals and commitments).

### 2.2 Using a Place‐Based Research Project to Advance Student Experiential Learning

Undergraduate student research fellows played a central role throughout all stages of this project while being co‐advised by a faculty member and Sustainability Office staff member (Table 2). Two students, supported by internally‐funded summer research fellowships sponsored by the faculty mentor, led key tasks including landscape mapping, data collection, ecosystem services modeling using i‐Tree, staff interviews, data synthesis, report writing and poster creation. The students independently mastered technical tools such as i‐Tree via publicly available online resources. Students worked with mentor faculty to define project scope and goals, including identifying pedagogical goals, which included in this case: developing research design skills, practicing interdisciplinary collaboration by interfacing with stakeholders, collecting and analyzing qualitative and quantitative data using interviews and ecosystem services analyses, synthesizing results from those analyses into recommendations, and presenting written and oral results to sustainability stakeholders and academic audiences (Table 2). If implementing a similar project, these pedagogical goals could differ in accordance with the needs of the “living laboratory” case study and students' professional development goals. This project was not embedded within a formal course or module but was pursued as an independent summer research experience. The initial project concept was broadly envisioned by the Sustainability Office staff mentor and faculty mentor but was further co‐designed collaboratively with the undergraduate student research fellows, who also engaged directly in stakeholder meetings with the Sustainability Office and Facilities Services teams as the project direction and scope were finalized. This approach could be transferred to different institutional contexts by developing a research/practitioner gap (in this case, the need to model the ecosystem services benefits of turfgrass conversion) *alongside* student mentees in order to co‐develop pedagogical aims that serve the needs and interests of those student mentees.

Student learning was supported through co‐mentorship by the faculty and Sustainability Office staff co‐mentorship. The students participated in weekly group meetings with the Sustainability Office student summer staff, which focused on applied sustainability work, and also participated in an additional weekly group “lab meeting” with the faculty member's summer research team. This combination engaged these students in both applied sustainability work happening on campus and a traditional summer research lab environment. Additionally, the two student fellows worked closely together on a daily basis and met weekly with their co‐mentors.

Products and deliverables included a report shared with campus stakeholders and academic posters presented both internally at a campus showcase and externally at a professional conference (the Ecological Society of America Annual Meeting). While no formalized learning outcomes or assessments were used (reflecting the project's rapid development as a COVID‐19 pandemic alternative after the cancelation of these students' original research opportunity) this experience offered training in research design, interdisciplinary collaboration, applied ecosystem science, and science communication. The students' leadership and initiative were crucial to the project's success, and conducting an experiential learning/living laboratory sustainability project via undergraduate research fellowship opportunities is a replicable model for integrating experiential learning outside of traditional coursework. Future iterations of this approach would benefit from strengthening formal pedagogical scaffolding and outlining learning goals to further enhance student professional development.

### 2.3 Determining Greener Future Land Use Scenario and Model Inputs

A campus tour with student research fellows and Facilities Services leadership was carried out to determine the following: the primary constraints to implementing sustainable land use management strategies, potential turfgrass land parcels that could be feasibly converted to native beds across campus, and resources necessary to transition to and maintain a sustainable landscape (e.g., mulch, labor, etc.). This provided context as to which types of landscaping would be better suited for the Macalester campus based on potential restraints.

Possible turfgrass parcels appropriate for conversion (“Greener Future” parcels) were identified after iterative collaboration between members of the Office of Sustainability and Facilities Services, including campus walking tours in which collaborating team members considered the aspect, hydrologic conditions, labor intensity needed in a given location to maintain lawn/garden beds/trees, and the social, recreational and/or educational benefits of keeping a given parcel as lawn (Figure 1a,b). This qualitative, iterative process led to the identification of parcels of turfgrass that could be converted to native plant beds and/or locations that could sustain the planting of new trees while also serving as a win‐win for Facilities Services. The Greener Future Scenario was ultimately defined as a conversion of 0.64 ha of turfgrass to native perennial gardens and planting of 1.99 ha of trees (Figure 1a–c).

**FIGURE 1:**
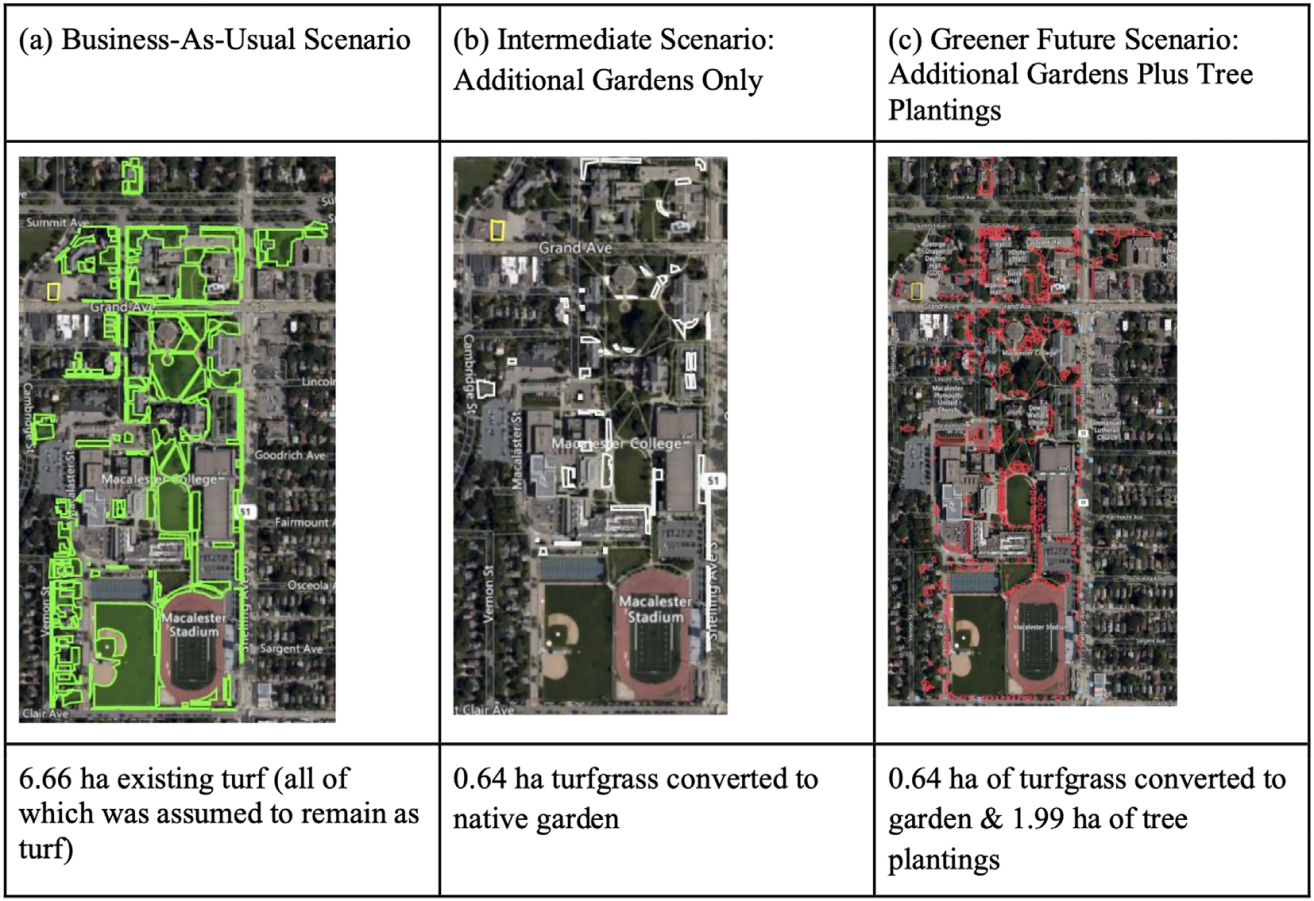
Business as usual and “Greener Future” scenarios for Macalester College Grounds. (a) Current land use and turfgrass (green outlines; 6.87 ha) map, Macalester campus; (b) low‐priority turfgrass areas (0.64 ha) converted to low‐input native gardens (white outlines) in “Greener Future” sustainable land‐use scenario; (c) low‐priority turfgrass areas (0.64 ha) converted to low‐input native gardens, plus overlay of suggested tree planting locations (10% increase in tree cover; 30% of total campus area tree cover is reached by planting 1.99 ha of trees) in “Greener Future” sustainable land‐use scenario (red outlines). Spatial extent measured using iLawn in conjunction with Macalester Facilities.

Undergraduate student research fellows also planned, designed and conducted a series of interviews with leadership in Facilities Services to determine what management practices were currently in use across the landscaping on campus and how management efforts differed between turfgrass parcels, native plant beds and trees (information needed for ecosystem services delivery modeling, see below). Guided interview questions can be found in Tour and Interview Question Guide, Data S1. These interviews included questions about fertilization practices, mowing schedules, budgetary constraints, irrigation, and pest and weed control.

### 2.4 Landscape Scenario Modeling

i‐Tree (www.itreetools.org) is a set of software modeling tools developed by USDA Forest Service scientists to enable practitioners to quantify the ecosystem services benefits delivered by trees and forests, and has been widely utilized to model urban trees (Nowak and Crane 2000; Wu et al. 2019; Lin et al. 2020, 2021). i‐Tree Canopy was used to map and quantify the current footprints of different land uses on campus, which was designated the Business‐As‐Usual (BAU) scenario. i‐Tree Canopy estimates land cover footprint by randomizing points over a satellite image of a landscape, which the user individually bins into land cover classes; using > 500 points is recommended to achieve a low standard error of the mean estimated area of each land cover class (Hwang and Wiseman 2020). Here, we use 700 randomized points overlaid on satellite imagery of Macalester's campus for landscape classification of the BAU scenario. Standard errors were generally low and there was no qualitative observation of clustering.

i‐Tree Canopy was then used to map and quantify the parcels chosen for conversion to native plant beds and for tree planting under a Greener Future (GR) scenario; parcels for conversion in the GR scenario were identified during the collaborative walking tour and iterative discussion between Sustainability Office student researchers and Facilities Services leadership described above. Macalester's campus currently contains approximately 20% tree cover. Official Macalester Landscape Management guides provide a tree cover goal of 50% to effectively reduce wind at ground level. We set an intermediate goal for this analysis of 30% tree cover in the GR scenario. The locations of additional trees to reach that intermediate goal were also collaboratively identified during the student/Facilities walking tour and iterative discussions.

i‐Tree Canopy requires input assumptions about the characteristics of pre‐defined land cover types. New and existing native plant garden beds were defined as 50% “landscaping shrubs/woody plants” and 50% “native prairie plants in a low biodiversity setting” (a mix of 9 showy prairie species). This assumption best reflects Macalester's current planting.

Several ecosystem services were quantified at the landscape scale as a model output, including services related to carbon storage, air quality, and water cycling. Each output reflects the total service delivered by Macalester's campus in either the BAU or GR scenarios. The modeled ecosystem services included (a) estimates of annual carbon sequestration rates in trees; (b) carbon stored in tree plant biomass; (c) annual removal of atmospheric CO, NO_2_, O_3_, PM10, PM2.5, and SO_2_; and (d) amounts by mass of avoided runoff, evaporation, interception, transpiration, potential evaporation and potential evapotranspiration.

While i‐Tree is widely used in urban forestry research and, especially, management (Nowak and Crane 2000; Wu et al. 2019; Lin et al. 2020, 2021; Hwang and Wiseman 2020), it is important to acknowledge its limitations. Among its advantages is that i‐Tree is freely available, relatively simple to learn, and requires low computational resources, making it well‐suited for implementation in living laboratory contexts such as this study where student involvement and ease of use are priorities. However, the underlying process‐based assumptions embedded in the models are relatively simple (facilitating low computational overhead). For instance, in our simplest ecosystem services estimates, used in this analysis, new tree cover was assumed to be planted exclusively over pervious surfaces without extending over impervious areas. This assumption directly led to the almost certainly inappropriate absence of measurable differences in hydrological metrics between the BAU and GR scenarios, a result that does not conform to real‐world conditions. While employing more complex aspects of the i‐Tree suite could better capture such effects, this introduces additional complexity and may reduce accessibility. In combination, the ecosystem service estimates presented in this study should be interpreted as model‐based approximations; though, outside of the hydrological services estimates, they are likely sufficiently accurate to guide sustainability management and planning at the institutional level.

### 2.5 Potential for Urban Fruit Trees on Campus

Students and Facilities staff were also interested in the potential for introducing more edible plants to the campus footprint. We thus explored the ecosystem service delivery of fruit‐bearing trees. Fruit trees on institutional lands have the potential to contribute to urban food forestry (Clark and Nicholas 2013; Wu et al. 2012), contributing to both urban food security goals as well as providing the ecosystem services delivery associated with many species of urban trees that do not produce edible food.

When building the GR scenario, three parcels that were identified as being suitable for conversion to tree planting were further earmarked for potential conversion to fruit trees. The chosen locations offered space for six trees in total (a subset of trees within the larger recommendation of increasing tree cover on campus to 30% cover) and were in central, high‐traffic locations on campus. Benefits were modeled for the following six fruit trees, as defined by i‐Tree: 2 common pear, 2 Honeycrisp apple, and 2 common plum, all of which grow well in Minnesota's climate (University of Minnesota Extension 2024a, 2024b, 2024c). Trees were modeled in pairs to allow for cross pollination. Once the three fruit tree plots were selected, i‐Tree was then used to model the benefits per tree, both monetary and environmental, from the year 2020 through the year 2038.

## 3 Results

### 3.1 Current Land Use (BAU) and Greener Futures Campus Sustainable Landscaping Plan

Macalester College manages 13.4 ha of grounds. The i‐Tree Canopy land cover analysis estimated that impervious surfaces (building footprints, roads/sidewalks, and a small amount of other impervious covers) and pervious surfaces (bushes, grass, gardens, soil/bare ground, and trees) account for 55.3% and 44.7% of total campus area respectively (Figure 2). From the same analysis, roads/sidewalks and building footprints both make up 22.0% ± 1.6% (mean ± standard error) of the coverage area, followed by tree coverage (20.8% ± 1.5%), grass coverage (18.4% ± 1.5%), other impervious surfaces (11.3% ± 1.2%), garden (4.0% ± 0.7%), bare ground (0.7% ± 0.3%), and bushes (0.7% ± 0.3%).

**FIGURE 2:**
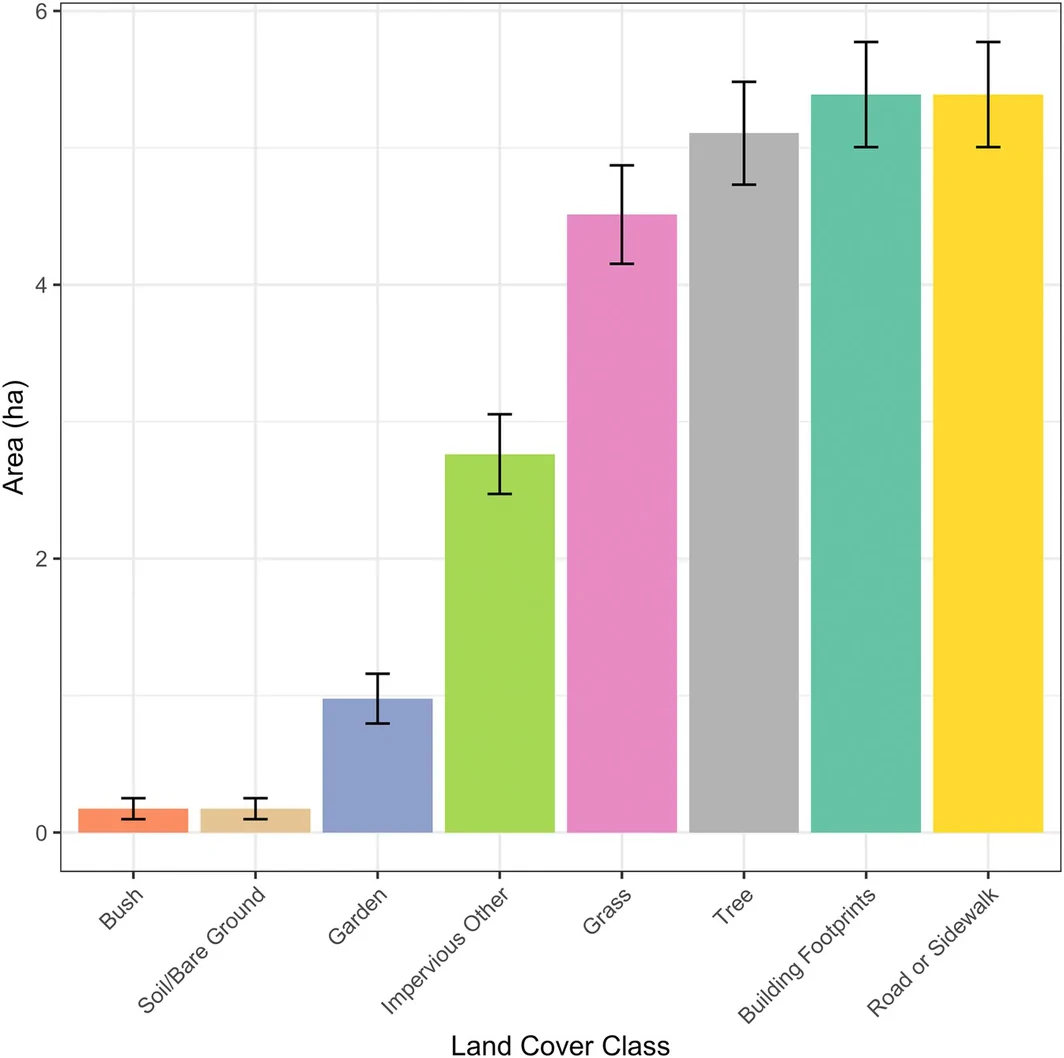
Current land use, Macalester College campus, reported as mean area (hectares) and standard error (error bars). Results from i‐Tree analysis. Impervious Other includes parking lots and other non‐road, non‐sidewalk, non‐building impervious surfaces.

Data sharing between the Office of Sustainability and Facilities Services further allowed for the collation of data (Tour and Interview Question Guide, Data S1) to show that the 2020‐era campus lawn footprint required 3175 kg year^−1^ of fertilizer, regular irrigation, and mowing efforts that emit 7.6 kg C year^−1^.

In the recommended Greener Future scenario, 49 parcels of low‐priority turfgrass areas account for 0.64 ha and were prioritized for conversion to low‐input native gardens and 1.99 ha of tree planting locations were identified, in comparison to the “Business‐As‐Usual” 2020 land use coverage (Figure 1); these parcels were identified manually in collaboration between stakeholders during campus walks. This scenario exceeds the intermediate goal of a 10% increase in tree cover across campus (to 30% of campus coverage) and instead represents the potential short‐term capacity for “win‐win” tree plantings. These 1.99 ha of additional trees would leave Macalester's tree coverage at approximately 36% of campus.

The Greener Future scenario also functions as a suggested sustainable campus landscaping plan, since it was designed in conjunction with Facilities Services and balances the availability of labor on the Facilities team. Installing new native plant beds is highly labor intensive in comparison to established lawn, but an established bed is much less intensive than a struggling lawn: after initial establishment of native beds, weed density decreases and the water and fertilization inputs decrease below that of an established turfgrass parcel. For instance, in a normal precipitation year, an established native bed will need very little to no supplemental water. For these reasons, struggling lawns were identified as priority areas to convert from turfgrass to native plant landscape beds.

### 3.2 Ecosystem Service Delivery Under Alternative Future Scenarios

The i‐Tree ecosystem services scenario analysis estimated that, if Macalester were to increase tree cover to ~30% campus area, convert 0.64 ha of lawn to low‐input native gardens, and follow Minnesota fertilization best practices, these changes, driven by an increase in tree cover, would sequester 21,744 kg of C per year, up from 16,193 kg of C per year, as well as increase the removal rate of all considered air pollutants (Table 3). This would result in an estimated increased human health benefit valued at $1034 year^−1^ (Table 3).

**TABLE 3:** Mean benefits in Business‐as‐Usual (BAU) vs. Greener Future (GR) scenarios over the course of a year.

	BAU	Greener future (beds plus trees)
C (trees) (kg year^−1^)	16,193	21,744
C (trees) (kg year^−1^, USD$)	$1522	$2044
C (trees) (kg, total on landscape)	406,645	546,031
CO (kg)	3.8	5.1
NO_2_ (kg)	28.5	38.2
O_3_ (kg)	215.5	289.4
PM10 (kg)	98.3	132
PM2.5 (kg)	10.1	13.5
SO_2_ (kg)	3.2	4.4
CO (kg, USD$)	$6	$8
NO_2_ (kg, USD$)	$21	$28
O_3_ (kg, USD$)	$883	$1186
PM10 (kg, USD$)	$679	$912
PM2.5 (kg, USD$)	$1427	$1916
SO_2_ (kg, USD$)	$1	$1
Avoided Runoff (L)	944,130	944,130
Evaporation (L)	3,392,192	3,392,192
Interception (L)	3,399,119	3,399,119
Transpiration (L)	6,935,066	6,935,066
Potential Evaporation (L)	28,521,072	28,521,072
Potential Evapotranspiration (L)	19,152,781	19,152,781
Avoided Runoff (L, USD$)	2229	2229

*Note:* Results from i‐Tree analysis.

Rainfall benefits are unchanged between i‐Tree ecosystem services models because all potential tree planting sites are currently over pervious cover, and trees will not immediately grow to overhang impervious surfaces (Table 3, discussion of i‐Tree advantages and limitations in Section 2).

Broadly, i‐Tree model results find that converting campus landscape from turfgrass to non‐turfgrass land cover enhances the capacity to manage for a more conservative nutrient cycle, higher carbon sequestration, and increased air quality. In this way, urban institutions may have broad‐reaching impacts on human health and urban ecosystems.

### 3.3 Potential for Urban Food Delivery via Fruit Trees

Over the course of 18 years, according to i‐Tree estimates, the six modeled fruit trees alone sequester roughly 2600 kg of CO_2_, with the apple trees sequestering 929 kg over that time period, the pear trees sequestering 1304 kg, and the plum 367 kg (Figure 3a). These trees would intercept 24,738 L of rainfall and help avoid 7147 L of stormwater runoff if placed such that they grow to overhang impervious surfaces (Figure 3b). There is a predicted removal of 1.4 kg of PM2.5 and O_3_. Changes in carbon cycle, water cycle, and air quality ecosystem services lead to a monetary value of over $10 year^−1^ for pear and apple trees by the time they reach maturity in the ecosystem services model estimates (Figure 3f). Tree maintenance utilizes less irrigation and fertilizer than traditional lawn, resulting in fewer yearly costs (savings of an estimated $18 USD year^−1^) in these i‐Tree estimates.

**FIGURE 3:**
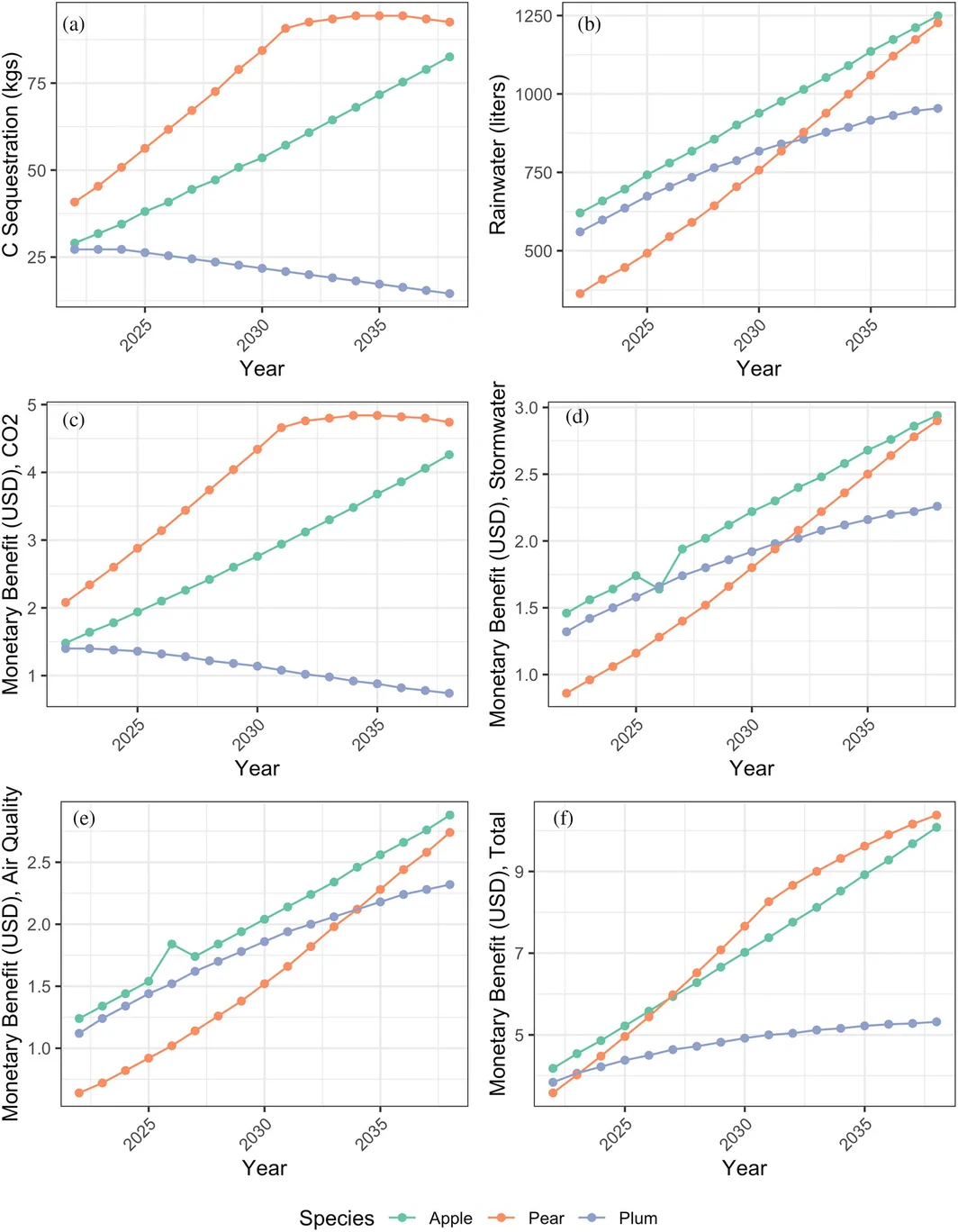
Ecosystem services benefits delivered over a 17‐year simulation by six fruit trees added to campus tree cover (two apple trees, two pear trees, two plum trees). Services measured include (a) carbon sequestered (in kg), (b) rainwater interception (in liters), (c) monetary benefits of CO_2_ sequestration (in USD), (d) monetary benefits of decreased stormwater runoff (in USD), (e) monetary benefits of air pollutant removal (in USD), and (f) total monetary benefits (sum of values in panel c–e). Modeling results from i‐Tree.

These findings suggest that sustainable land changes/practices can increase a campus' CO_2_ sequestration while also improving air quality and reducing rain runoff. Although low in monetary benefit, this strategy can help colleges reach both sustainability and carbon neutrality goals.

## 4 Discussion

A silver lining of the COVID‐19 pandemic was that it brought together a group of campus stakeholders whose spheres of work on campus do not normally overlap. This group discovered they shared an interest in reducing turfgrass on our campus and were able to find a shared project goal that aligned with all of their interests as well as met their constraints.

### 4.1 Recommendations for Macalester Campus

This team's i‐Tree modeling shows that there are significant ecosystem services to be gained from planting native gardens and trees on Macalester's urban campus. In addition to the ecosystem services modeled here—namely, ecosystem carbon storage, water cycle regulation, and air quality improvement—additional ecosystem services benefits, including decreased energy usage and a more conservative N and P nutrient cycle, will help Macalester fulfill institutional sustainability goals. Our findings also suggest that there are numerous win‐wins on Macalester's campus that would successfully balance the fiscal constraints placed on Macalester's Facility Services team, the need for turfgrass areas for college social goals, and the college's desire to advance sustainability goals on campus.

Specifically, our findings led to several recommendations to the Macalester Facilities Services team:

- Convert 6381.8 m^2^ of unused or struggling/low‐quality turfgrass to low‐maintenance native garden beds.
- Reduce the use of phosphorus fertilizers on campus turfgrass parcels and reduce the overall fertilizer addition on campus sports fields.
- Continue to plant trees, with a long‐term goal to reach ~50% tree coverage over time, with an intermediate goal of ~30% tree coverage.

There are numerous mutually beneficial outcomes associated with these recommendations. Converting struggling turfgrass parcels to native garden beds reduces labor needs after beds have been established (e.g., no mowing, reduce watering, minimal fertilizing), which increases efficiency for the Facilities Services team, and also adds landscape to campus that is pollinator friendly, filters rainwater, and is esthetically pleasing, which fits with the Sustainability Office and Campus strategic goals. Reducing fertilizer usage will likely lower campus greenhouse gas emissions via a reduction in nitrous oxide emissions and decrease the N and P loss via runoff and leaching. Finally, planting additional campus trees also helps meet both Facilities Services goals and Sustainability goals (labor efficiency, lowering long‐term costs, increasing campus ecosystem service delivery).

These results are consistent with a long history of research showing that urban land management decisions can have wide‐ranging ecosystem services benefits. Increased tree cover has been linked to overall increases in community health and reduced crime rates (Eisenman et al. 2019). Urban trees reduce N and P leaching to groundwater, helping manage for a more conservative nutrient cycle (Nidzgorski and Hobbie 2016) as well as sequester carbon and decrease air pollution. High levels of tree cover can also provide shading and passive heating which may then allow for reduced energy use for structures (Fissore et al. 2012).

Notably, these recommendations do not include the elimination of all lawn or turfgrass, which have unique utility on college campuses and similar institutional parcels of land. They are required for athletics and are also crucial as gathering spaces—turfgrass lawns provide numerous recreational spaces that live at the heart of many residential college campuses. These recommendations indeed only include eliminating ~10% of the current footprint of lawns on Macalester's campus.

### 4.2 Legacy of Turfgrass Removal Research and Cross‐Campus Sustainability Collaboration

This project led to an ongoing collaboration between the Sustainability Office, Facilities, and students. Since this research project was implemented, the Macalester Grounds team has continued to work on converting turfgrass to native plant beds on campus. In the 5 years since the analysis reported here, Macalester has converted approximately 492 m^2^ of turf to urban meadow, prairie garden, and pollinator garden. Macalester has also begun experimenting with bee lawns and has added 239 m^2^ of clover area to existing turf. In addition, the Macalester grounds crew has planted 21 additional trees throughout campus (including a variety of species from large native shade trees to ornamentals). There are more tree planting and turf conversion projects in the works, and the faculty and staff that have been involved with these projects are continuing to identify opportunities for student‐led research. The success of this project and the continued work that has been built upon it suggest that similar initial case studies can lead to meaningful, impactful, and sustained collaborations across departments and groups on college campuses.

### 4.3 Implementation Best Practices and Ongoing Work

Several best practices have helped us to maintain the benefits generated by this project. These practices include:

#### 4.3.1 Early Cross‐Department Communication and Collaboration

Early and sustained communication, collaboration, and compromise between campus departments were key to the success of this project and the continuation of the benefits that it generated. In particular, regular conversations with Macalester's Lead Groundsperson ensured that this project produced results and recommendations that were both theoretically relevant, practically impactful, and implementable. For example: It was important to pay close attention to the constraints faced by Grounds when identifying areas for turfgrass conversion. Thus, student researchers explicitly discussed the use and maintenance of different sections of turf on campus with Grounds. The Lead Groundsperson was able to highlight both areas where turf was very commonly used and could not be removed as well as areas that were strong candidates for turf conversion. Areas that were highlighted as having the best potential for conversion included parcels that were rarely used, were difficult to maintain as lawn, and did not have any irrigation or grading challenges. By focusing upon this low hanging fruit, we were able to identify areas where turfgrass conversion would maximize the mutual benefits of maintenance efficiency and ecosystem benefits that we were seeking with the project.

#### 4.3.2 Connect Theory to Practice

Through its design, this project also highlighted the connection between sometimes abstract theory and real‐world practice. Student enthusiasm for the project was bolstered by the idea that their work could lead to real‐world campus sustainability progress. It also provided a learning opportunity for students to see how academic research can be translated to hands‐on practice. At the same time, the data‐driven results helped to support Facilities staff and Sustainability Office efforts to promote turfgrass conversion. Subsequent projects that came about as a result of this project have provided continuing opportunities for student research. For example, subsequent (and currently ongoing) research projects focusing on measuring the impacts of turfgrass conversion and rain garden projects on water quality provide additional opportunities for hands‐on research linked to real‐world sustainability efforts being implemented by the Macalester Sustainability Office and the Grounds teams.

Another aspect of this project that made it particularly impactful from a research, education, and implementation standpoint is that it was connected to existing sustainability goals. By connecting to existing campus plans, the results of the project were seen as supportive to work being done on campus rather than an additional item that would add to already large workloads. This has helped Macalester to maintain the momentum generated by the project well past its end date and ensure that the recommendations that came out of the project were implemented.

#### 4.3.3 Provide Support for Implementation

The Macalester Facilities Services Department and the Sustainability Office have collaborated to identify financial support for continued tree planting and conversion projects. Many of the new trees on campus have come as memorial donations, which keeps the cost of the trees out of the grounds budget. In addition, Macalester has received native plant donations and applied for and received several grants to pay for continued tree planting and turfgrass conversion projects. Some of the funding has come through collaboration with the local soil and water conservation district, which has helped the college access state and federal funding for projects that will support regional stormwater management goals. Grounds and the Sustainability Office also received a $10,000 grant from the college to plant additional trees and organize educational events around campus tree cover for Macalester and the surrounding community as part of the college's sesquicentennial anniversary (or “anni‐fir‐sary” as the project team is calling it).

It is also important to recognize that the process of turfgrass conversion can initially be labor‐intensive. Turf removal must be relatively deep to remove turfgrass roots and prevent reestablishment. Once turf is removed, the area is prepared for planting though scarification and/or by adding topsoil. Once the new beds are planted, the area is heavily mulched to help prevent weeds and aid the restoration of topsoil. Finally, over the first 3–5 years after planting, native plant beds require routine weed removal, hand watering, and re‐mulching. Once beds are established, the maintenance needs are reduced. However, it is important to provide additional support for maintenance during the first years of the conversion projects to ensure their success.

The Grounds team has collaborated with the Sustainability office and student groups on campus to help with maintenance needs. Several student groups on campus and the Sustainability Office staff have led well attended volunteer events to support planting new pollinator beds. Two student‐led turfgrass replacement projects have taken place since 2020, and more are in planning. This continued collaboration with students has been beneficial to Grounds staff. With the help of students and the Sustainability Office, Facilities has been able to address the roadblock of lack of available labor and care for existing pollinator beds, other turfgrass conversion plantings, and future conversion projects. To continue this work, the Grounds team and Sustainability Office have collaboratively sponsored student workers and are currently working on developing a student worker position that would help to plant native seedlings in the campus greenhouse for use on campus grounds, facilitate the use of native plantings in landscaping, organize planting events for turf conversion projects, and help with maintenance during the first few years of new plantings.

#### 4.3.4 Embrace the Opportunity to Use the Campus as a Living Laboratory for Hands‐On Experiential Learning

College campuses occupy a special sort of institutional space: they include spaces for learning, but also recreation, housing, office work, and community gathering, and have the opportunity to be a “living laboratory” for students (Hansen 2016; Lindstrom and Middlecamp 2017; Zen 2017). Macalester, like many residential colleges, places value on experiential learning (Macalester College 2024). Several campus classes focus on or engage directly with the grounds, led by faculty and staff in the Environmental Studies and the Sustainability Office (Hansen 2016). There is an opportunity to build upon these opportunities through offering additional courses and continuing to engage faculty, including in Biology, Environmental Science, and food‐ and agriculture‐related courses. This learning modality need not be limited to environmentally focused courses. The campus grounds can provide opportunities for students in courses from mathematics to education to creative writing to gain experience in topics such as modeling, community education, and nature writing (Favaloro et al. 2019; Sundman et al. 2025).

Campus grounds provide opportunities to expand learning opportunities beyond students to faculty, staff, and community members (Gunnels et al. 2021; Parr and Trexler 2011). Areas such as the pollinator path, campus rain gardens, and native‐plant prairie include interpretive signage which engages and educates the community. There are well‐attended landscape tours of the campus each year directed by Facilities Services aimed at faculty, administrators, and staff that help the community connect with their environment. The Sustainability Office and Grounds Department are currently collaborating to develop a tree tour of campus with additional interpretive signage to highlight tree species on campus. These opportunities can be recreated at college campuses writ large, regardless of biome or the precise nature of the sustainable campus landscaping initiatives.

Finally, a crucial aspect of this project's success was the fact that undergraduate student researchers were both empowered to take on leadership around a living laboratory/experiential learning sustainability effort and that internal campus resources (i.e., funding for summer undergraduate research fellows) were available to support those student efforts. Students were also concurrently provided with an opportunity for professional development that could be replicated at similar institutions where undergraduate research experiences outside of a structured coursework environment are highly valued. This dynamic was a two‐way “win‐win” in sustainability and environmental science education, in which a co‐curricular experience benefitted students and campus sustainability goals and interdisciplinary collaboration was advanced because of the leadership and efforts these students provided.

## 5 Conclusion

The results of this modeling exercise advanced campus sustainability goals and served as a case study in academic practice by embedding student‐driven experiential learning within an interdisciplinary, place‐based research collaboration. The project fostered a valuable interdisciplinary collaboration at Macalester College, bringing together campus stakeholders, including undergraduates, from various departments who shared a common interest in increasing the ecosystem services and the sustainability potential of Macalester's grounds. This collaborative effort resulted in recommendations to replace strategic parcels of turfgrass with native plant beds and increase campus tree cover and has since blossomed into a robust collaboration between Facilities Services' Grounds team, the Sustainability Office and student organizations that led to the addition of numerous trees and plant beds on campus. The success of this project underscores the opportunities present in bringing undergraduate researchers into cross‐departmental sustainability collaboration and providing experiential learning opportunities for students to connect theoretical research to real‐world applications. The ongoing projects and student‐led research initiatives illustrate the value of using the campus as a living laboratory. This case study serves as a model for other institutions seeking to strengthen the connection between sustainability objectives, experiential learning and undergraduate mentorship.

## Author Contributions

**Christine S. O'Connell:** conceptualization (equal), formal analysis (equal), funding acquisition (equal), project administration (equal), writing – original draft (equal), writing – review and editing (equal). **Shannon Hahn:** conceptualization (equal), data curation (equal), formal analysis (equal), investigation (equal), methodology (equal), writing – review and editing (equal). **Evelyn Jauregui:** conceptualization (equal), data curation (equal), formal analysis (equal), investigation (equal), methodology (equal), writing – review and editing (equal). **Michael V. Frazier:** conceptualization (equal), data curation (equal), methodology (equal), writing – review and editing (equal). **Anika R. Bratt:** formal analysis (equal), validation (equal), writing – original draft (equal), writing – review and editing (equal). **Mary A. Heskel:** formal analysis (equal), validation (equal), writing – original draft (equal), writing – review and editing (equal). **Megan Butler:** formal analysis (equal), validation (equal), writing – original draft (equal), writing – review and editing (equal). **Christie M. Manning:** conceptualization (equal), data curation (equal), formal analysis (equal), investigation (equal), methodology (equal), project administration (equal), supervision (equal), writing – original draft (equal), writing – review and editing (equal).

## Conflicts of Interest

The authors declare no conflicts of interest.

## Supporting information


**Data S1:** Question guide for sustainability students to use when interfacing with facilities/landscaping teams.

## Data Availability

Data and code associated with this manuscript are available from the Dryad data repository at https://doi.org/10.5061/dryad.dv41ns2bq.
