# Supplementary material for: Sustainable Landscaping and Sustainability Education on a College Campus: A Case Study in Place‐Based, Collaborative Ecosystem Science
Source: Ecol Evol. 2026 Oct 11;16(10):e74486. doi: 10.1002/ece3.74486 (PMC13654617; doi:10.1002/ece3.74486)
Supplement: Supplementary file 1 [file ECE3-16-e74486-s001.pdf]

Supplemental Document 1: Question guide for sustainability students to use when interfacing with facilities/landscaping teams

“Zones”

How much land overall does Facilities manage (how many acres?)

- On the Saint Paul campus
- Outside of the Saint Paul campus (e.g., the Ordway Field Station, houses and businesses owned by Mac)

How are the macalester grounds divided up (are there different “zones” like shaw field, the flower bed in front of Weyerhaeuser, sidewalks, courtyards, ect?)?

What management tasks are performed in each zone?

How often are they performed?

Who performs them?

Economic

Is there a master spreadsheet of maintenance costs per month/year?

Facilities/ Grounds crew schedule and budget for landscape maintenance?

How much does fertilizer cost? Do you buy in bulk? How much and how often?

What are the costs associated with maintaining the landscape?

Machinery?

Water?

Student and staff employment (number of students and staff employed, how much they work each season)?

Frequency of these expenses?

Initial planting costs?

Area of each “zone”

How large is each rain garden?

How large is the prairie?

How large is the pollinator path?

Is there an up to date map of the grounds that is to scale and shows the different grounds areas (e.g., rain garden, planting beds, lawns, flower beds, etc.)?

Planting/ species map

Is there a map showing which plants have been planted where and when?

Is there a list of all the tree and plant species on campus?

Mowing
What is the mowing schedule?
Which areas are mowed?
Who is in charge of/performs mowing?
What types of mowers are used for each area?
What happens to yard clippings?
How is bare soil treated?

Pest control
What pesticides/herbicides/insecticides (brands) are used?
- For weeds
- For insects
- For animal pests

How often and in which “zones”?
What quantity of each pesticide is used?

Watering
What is the watering schedule (how often and what time of day)?
Which areas are watered?
What types of irrigation are used?
What type of irrigation for each zone (eg sprinklers, drip, none)?
How much water is used, in gallons per week throughout the season for each irrigation type and zone?
Do we have water storage or gray water holding capacity?
Do we use any non-potable water for irrigation?

Fertilizing
How often is fertilizer used? And in what amounts is it applied?
Fertilizer brand(s) and forms (urea, nitrate, etc)?
Where is each fertilizer used? (eg whole campus or only sports field)
How often are campus lawns fertilized?
Fertilizer application rate (both per fertilization event and per year)?
What is the NPK value of Macalester’s fertilizer?
Fertilization timing (e.g., before/after mowing/watering/rain/night?)?

What are the fertilizer practices for plots of lawn with mainly trees planted on them?

Other

What yearly activities are done to prepare for and end the growing season?
What type of ice melts are used, in which areas or conditions, and in what proportions in winter?
Is there an estimate of the total weight used?
What types of machinery are used to perform groundworks?
Are there storm drains on campus? If so, where?
